# Longitudinal Analysis of Variability in Fecal Glucocorticoid Metabolite Concentrations in Three Orangutans (*Pongo pygmaeus pygmaeus* and *Pongo pygmaeus abelii*) before, during, and after Transition from a Regular Habitat Environment to Temporary Housing in Indoor Holding Facilities

**DOI:** 10.3390/ani12233303

**Published:** 2022-11-26

**Authors:** Laurel B. Fink, Asaba Mukobi, Lindsey Gruber, Colleen Reed, Jason DeLibero, Scott Jackson, Sierra Neill, Julia Walz, Cydney Sines, Becca VanBeek, Candace D. Scarlata, Nadja Wielebnowski

**Affiliations:** 1 Oregon Zoo, Portland, OR 97221, USA; 2 Department of Biology, Portland State University, 1719 SW 10th Avenue, SRTC rm 246, Portland, OR 97201, USA

**Keywords:** zoo animal welfare, wildlife endocrinology, stress, fecal glucocorticoid metabolites, orangutans, transfer stress

## Abstract

**Simple Summary:**

Between May 2018 and December 2020, three orangutans (*P. p. pygmaeus* and *P. p. abelii*) were housed at a holding facility (Veterinary Medical Center, VMC) during an extensive period of construction of a new primate habitat at the Oregon Zoo. Over 1400 fecal samples were collected and analyzed to quantify adrenal responses of orangutans while housed in the VMC holding area versus their original habitat (Red Ape Reserve, RAR). Using a previously validated corticosterone enzyme-immunoassay (CJM006), fecal glucocorticoid metabolite (fGM) concentrations were monitored to track adrenal activity across three time periods: the initial housing at the RAR (RAR1), temporary housing at the VMC (VMC), and the return to RAR (RAR2). The results showed significantly elevated fGM concentrations in samples collected at the VMC when compared to RAR2 for orangutans “B40236” and “1149”; and when compared to RAR1 for orangutan “1149”. However, the actual transfers between the different types of housing did not result in a significantly elevated fGM peak for any orangutan. This finding may provide some indication that intensive pre-transfer training by animal care staff potentially helped to alleviate stress responses to intra-institutional transfers. Overall, our study highlights the importance of monitoring intra-institutional transfers and responses to temporary housing with the same scrutiny that is increasingly being applied to inter-institutional transfers in order to identify best practices and response.

**Abstract:**

Considerable research has been conducted on the effects of inter-institutional transfers, but far less consideration has been given to intra-institutional transfers and extended housing in off-habitat holding. On 15 May 2018, The Oregon Zoo’s orangutans (*n* = 3) were moved from the Red Ape Reserve (RAR) to the Veterinary Medical Center (VMC) indoor holding areas and remained there until 22 December 2020, resulting in over two years of housing in a facility not specifically designed for orangutans. This study aimed to quantify potential changes in fecal glucocorticoid metabolites (fGM) typically associated with increased adrenal activity as a result of transfers, as well as potential differences in fGM concentrations associated with housing in the two different types of locations. We collected fecal samples from all orangutans during three time periods: the initial housing at RAR (RAR1), the time spent at VMC holding (VMC), and the return to RAR (RAR2). Samples were analyzed using enzyme-immunoassay (EIA) analyses and compared using two-way ANOVA tests with Games–Howell post-hoc evaluations. The results of our analyses showed the following: (1) significant differences in fGM concentrations based on location in two orangutans, with the highest fGM concentration occurring in fecal samples collected at the VMC; and (2) a lack of significant fGM peaks following multiple intra-institutional transfers for all three orangutans. Though requiring further corroboration through future studies, we speculated that pre-transfer behavior training and intensive, continued care by familiar animal care staff may have helped to mitigate the stress responses commonly associated with transfers and major changes in housing. Furthermore, this study highlights the individualistic nature of the stress response, as illustrated by the substantial variation in fGM concentrations across different housing regimens in the three orangutans.

## 1. Introduction

In the zoo environment, animals may often experience both intra- and inter- institutional habitat transfers due to breeding recommendations, dispersal requirements, construction, or other necessary disruptions. Construction of new animal habitats and/or extended maintenance needs of older habitats may require temporary intra-, or even inter-, institutional relocation of affected individuals. Typically, inter-institutional transfers receive considerable scrutiny in terms of animal welfare monitoring, while intra-institutional transfers have been, thus far, less studied. However, when examined, both intra- and inter-institutional transfers have been found to lead to considerable elevations in fecal glucocorticoid metabolite (fGM) concentrations in multiple species (e.g., *Prionailurus viverinus* [1], *Varecia variegata* [2], and *Elephas maximus* [3]). A trade-off has to be made as to whether it is feasible to translocate animals temporarily within the institution, and thus lower overall transport stress, or send them out to other institutions until construction has been completed. In some cases, the latter option may not be feasible depending on the particular species and/or individual requirements due potential lack of availability of appropriate enclosure space at other accredited institutions.

At the Oregon Zoo, construction of a new primate facility resulted in a prolonged, yet temporary, intra-institutional relocation of all orangutans (1.1 Bornean orangutans, *Pongo pygmaeus pygmaeus* and 0.1 Sumatran orangutan, *Pongo pygmaeus abelii*). On 15 May 2018, the orangutans were moved from the Red Ape Reserve (RAR) habitat into the Oregon Zoo’s Veterinary Medical Center (VMC) holding facility and the Zoo’s chimpanzees were moved into RAR temporarily to accommodate the demolition of the old, and construction of a new chimpanzee facility. The initial anticipation for this temporary relocation was a much shorter time period, but due to construction delays the orangutans remained at the VMC until December 2020, resulting in more than two years at the holding facility. While the temporary housing of individuals at the VMC is not uncommon, the two-year duration is unusual. This unique situation provided an opportunity to observe how long-term housing in temporary indoor holding facilities, and the associated intra-institutional transfers may have affected the adrenal activity of orangutans at the Oregon Zoo.

Previous research on orangutan adrenal activity in response to transfers identified some strong trends in physiological expression and helped to inform this study. For example, inter-institutional transfers resulted in significant peaks of fecal glucocorticoid concentrations within 24–48 h after transport in both wild-living [4,5] and captive-living orangutans [5,6], and a study using data from the International Orangutan Studbook found a high rate of mortality in orangutans within the first-year post-inter-institutional transport [7]. It appears that, so far, no formal studies have been conducted on the adrenal responses of orangutans transferred to temporary housing facilities within the same institution and their subsequent acclimation to those facilities. However, a recent study conducted on three species of lemur (*Lemur catta, Eulemur albifrons,* and *Eulemur collaris*) showed increased frequencies of elevated fGM concentrations in the latter-half of a 30-day period when the lemurs were housed in temporary facilities [8]. 

Both the Association of Zoos and Aquariums (AZA) and the European Association of Zoos and Aquariums (EAZA) outline specific guidelines for inter-facility transport, including pre-shipment veterinary evaluations, quarantine guidelines, and behavioral training to minimize the impact of crating and other transport-related stressors [9,10]. Yet when animals are being transferred within the same facility very limited, if any, guidelines are available for such moves. This is, in part, due to the fact that animals are not changing ownership and are not travelling outside the institution, thus not requiring any additional “third party” transport services and associated considerations. Nevertheless, especially when large and/or potentially dangerous mammals are involved, animal care managers and staff will commonly develop detailed internal protocols and pre-transfer training plans specifically designed to maximize safety, minimize stress, and minimize the overall impact of intra-institutional transfer/relocation on animals and staff.

Our study aims to quantify how intra-institutional transfers and prolonged temporary housing in an indoor holding facility impacted the adrenal physiology of the orangutans at the Oregon Zoo. Due to the information presented in previous research (e.g., the increased frequencies in elevated fGM concentrations over time at temporary holding facilities in lemurs [8]), and the fact that the orangutans would be returning to their previous enclosure (RAR) after the time spent in indoor holding (VMC), we predicted that the average fGM concentrations of the two species, *P. p. abelii* and *P. p. pygmaeus*, would likely increase while the orangutans were housed at the VMC and decrease when they returned to the familiar RAR. Furthermore, based on previous findings [5,6] on how other orangutans have reacted to institutional changes and transport itself, we predicted a significant peak of fGM concentrations in direct response to the crated transport across the Zoo to a different location. 

## 2. Study Animals and Methods

### 2.1. Animals and Facilities

Two captive born orangutans (1.1 *P. p. pygmaeus*) and one wild-born orangutan (0.1 *P. p. abelii*) housed at the Oregon Zoo (Portland, Oregon) were included in the study (Table 1). All orangutans were fed a mixture of Mazuri Primate L/S Biscuit Banana (5M1G) and fresh fruit and vegetables four times per day through scatter-feeding methods. Individuals were originally housed at RAR, a large indoor and outdoor facility (3212 sq. ft.) specifically designed for this species. The orangutans were given outdoor access when the temperatures exceeded 32 °F (0 °C). On 15 May 2018, all orangutans were moved to the VMC, an exclusively indoor environment containing five different enclosures (each ranging between approximately 110 sq. ft. and 175 sq. ft.) and connected via gates that could be manipulated by care staff. Although the VMC only provided an indoor holding area, there were skylights that allowed for exposure to natural sunlight. The orangutans were transferred back to RAR on 22 December 2020. On 13 January 2021, two white-cheeked gibbons (1.1 *Nomascus leucogenys*) were brought to RAR where they were allowed visual, olfactory, and auditory access to the orangutans until they were introduced on 30 April 2021. Thereafter, the gibbons were housed with orangutans.

The three orangutans were usually housed together but were managed individually if social conflicts occurred and to support the fission-fusion social structure of wild orangutans. This management style has been shown to reduce potential social stress [11].

### 2.2. Sample Collection and Steroid Extraction 

All three orangutans were included in the fecal glucocorticoid metabolite (fGM) monitoring. For this study, fecal collection occurred at least 3x per week between 26 March 2018, and 30 April 2021. Due to the existence of diurnal fluctuations in orangutan fGM concentrations [5], fecal collections were limited to defecations collected between 7:00 a.m. and 10:00 a.m. during routine cleaning. For orangutan “1149”, fecal collection stopped on 6 January 2021, when she was humanely euthanized due to decreasing quality of life associated with old age (at close to 61 years of age this individual was the oldest orangutan documented in North America according to Studbook data). Introductions to new individuals have been shown to elevate fGM concentrations in apes (*Gorilla gorilla gorilla* [12]); therefore, the decision was made to end fGM analysis for “B50023” and “B40236” prior to the introduction of the white-cheeked gibbons on 30 April 2021. 

Care staff were instructed to avoid samples contaminated by urine as glucocorticoid metabolites are secreted in both urine and feces but to maintain consistency, only one medium should be analyzed at once [13,14]. Food-safe dyes (Soft Gel Paste Food Color, AmeriColor, Placentia, CA, USA) delivered to the orangutans in daily dietary items by care staff were used to stain the fecal samples and assist in identification of the defecations (see [15] for a review of fecal markers). Each fecal sample was placed in a re-sealable plastic bag and immediately frozen at −4 °F (−20 °C) until analysis. Steroid extraction methods were adapted from methods used by Bryant and Wielebnowski [16]. Briefly, 5 mL of 80% methanol was added to 0.5 g (±0.025 g) of homogenized wet fecal matter, vortexed, and shaken overnight for 17 h (open air rocking shaker, Fisherbrand^TM^). Samples were then centrifuged (ST 16, Sorvall^TM^) at 2500 rpm for 15 min. An amount of 3 mL of the resulting 1:1 supernatant was removed and 500 µL were desiccated in a SpeedVac (Savant Speedvac DNA110) before being reconstituted in 500 µL Tris HCl assay buffer (Arbor Assay, Ann Arbor, MI, USA). Samples were further diluted to 1:8 in Tris assay buffer before analyses for glucocorticoid metabolites.

### 2.3. Enzyme-Immunoassay (EIA)

**Figure 1:**
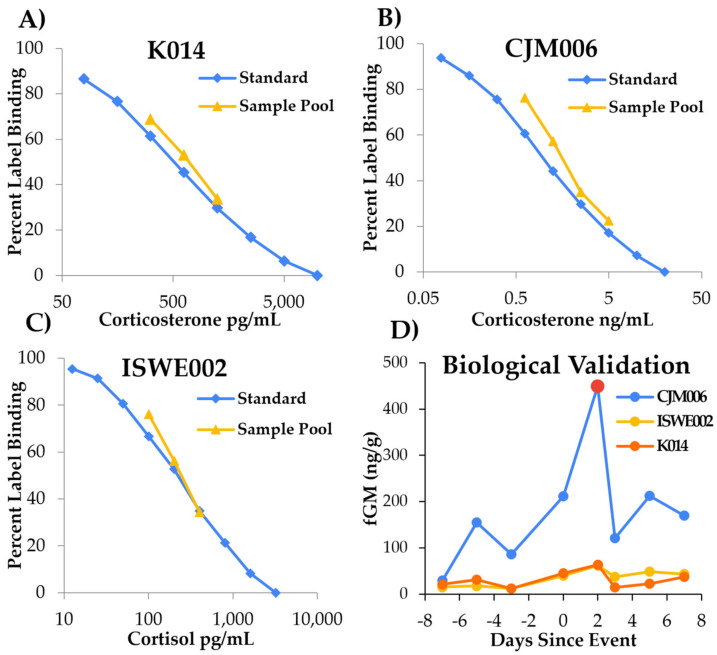
Orangutan fecal glucocorticoid metabolite (fGM) validations. (**A**) Corticosterone EIA #K014 (Arbor Assay), (**B**) Corticosterone EIA CJM006 (C. Munro, University of California, Davis), (**C**) Cortisol EIA ISWE002 (Arbor Assay), (**D**) Biological fGM validation. Data greater than 2 standard deviations above the mean considered significantly elevated (•).

It has previously been determined that orangutan fecal glucocorticoid metabolites (fGM) can be reliably monitored and fGM assays are biologically valid [4,5,6]. To demonstrate validity in our own lab, three separate enzyme-immunoassays (EIA) were tested for chemical and biological validation at the Oregon Zoo Endocrine Lab and Welfare Monitoring Program. Chemical validation was provided by showing parallelisms between binding inhibition curves of orangutan fecal extracts and the standard curve for the corticosterone assay. Significant increases in fGM concentrations in response to a known strong stressor (in this case, a social conflict resulting in injury) were used for biological validation. Both corticosterone EIAs were successfully validated for orangutan fGM tracking: #K014 (Arbor Assays) (Figure 1A) and CJM006 (C. Munro, University of California, Davis) (Figure 1B). The cortisol EIA kit (ISWE002) exhibited the least successful chemical validation (Figure 1C). For biological validation, a significant elevation in fGM concentrations (>2 standard deviations above baseline) was observed 48 h after the observed social conflict and injury for all three assays, but the CJM006 assay proved to be the most sensitive and provided the highest concentration. Unfortunately, fecal samples were not collected 24 h after the social conflict, but rather 48 h; therefore, the measured fGM elevation may have started earlier than observed. However, the results in our study were similar to findings by Weingrill et al. [6] who measured peak fGM concentration between 48 and 72 h after translocation.

Due to the successful biological validation and chemical validation along with the increased sensitivity of the assay (Figure 1D), fGM concentrations were monitored using the CJM006 EIA. A polyclonal antibody produced against corticosterone-3-CMO-BSA (CJM006; 1:100,000; C. Munro, University of California, Davis), a horseradish-peroxidase conjugated corticosterone label (1:100,000) and corticosterone standards (0.078–20.0 ng/mL) was used in the EIA. The 96-well microtiter plates were pre-coated with secondary goat anti-rabbit IgG antibody (150 µL/well at 0.10 mg/mL, A009, Arbor Assays, Ann Arbor, MI, USA) using a process developed by Arbor Assays (ISWE Plate Coating Course). The assay sensitivity was 0.100 ng/mL with inter- and intra-assay coefficients of variation (CVs) maintained below 15% and 10%, respectively. This corticosterone antibody cross-reacts 100% with corticosterone, 14.25% with desoxycorticosterone, 2.65% with progesterone, 0.90% with tetrahydrocorticosterone, 0.64% with testosterone, 0.23% with cortisol and <0.10% for five other steroids tested (C. Munro).

### 2.4. Data Analysis

All statistics were analyzed using R-Statistical package (version 4.0.3; [17]). Baselines were calculated using the package “hormLong” [18]. Based on our findings and in accordance with Weingrill et al. [6], we identified a significant peak in fGM concentrations at 48 h post-stressor. Sample collection dates were therefore backdated by two days to account for this lag time. Additionally, to account for the difference in subspecies and substantial differences in fGM concentrations observed in orangutan “1149” when compared to the other two individuals, all analyses were conducted twice: once including data from orangutan “1149”, and once excluding her data. 

#### Habitat and Individual Differences

To compare chronic glucocorticoid changes associated with changes in habitats, the glucocorticoid concentrations were averaged over the time pre- (“RAR1”; 26 March 2018–16 May 2018), during (“VMC”; 17 May 2018–22 December 2020), and post- (“RAR2”; 23 December 2020–28 April 2021) habitat transfer for each orangutan. As introductions to new conspecifics are known to elicit a strong stress response in apes [12], the analysis ended immediately prior to the introduction of a pair of white-cheeked gibbons to the orangutans at the RAR on 30 April 2021. Both overall and baseline averages were calculated and compared using the package “hormLong” [18]. Baseline means were calculated using an iterative method in which values greater than two SD above the mean were excluded and the means were recalculated until all values greater than two SD above the mean were eliminated [19,20]. For all other analyses, data were log-transformed to meet the assumption of normality. Normality of the log-transformed data was determined by visual assessments of Q–Q plots of residuals and relevant histograms. As a Levene’s test revealed unequal variances, and the sample collections were unbalanced at each habitat, type one (hierarchical), two-way ANOVA tests were conducted using weighted means, while Games–Howell post-hoc tests were used to compare the effects of habitat location (“Location”) and individual animal (“Focal”) on the fGM concentrations [21]. Since it is well-known that individuals within the same species can express statistically significant differences in fGM concentrations under the same conditions [22,23], the hierarchical ANOVA computation was used to place higher importance on “Location”. All data were reported as back-transformed mean ± standard deviation (SD) of the mean, unless otherwise stated. 

## 3. Results

### 3.1. General Descriptive Data

Between 26 March 2018, and 28 April 2021, over 1400 fecal samples were collected and analyzed (B50023, *n* = 506; B40236, *n* = 505; 1149, *n* = 426) and both baseline and overall descriptive data are presented in Table 2. 

### 3.2. Habitat and Individual Differences

**Figure 2:**
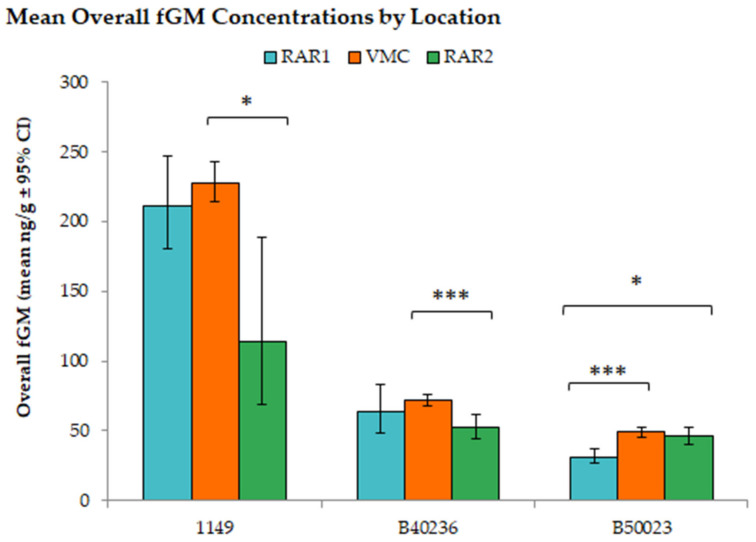
Average overall fGM concentrations (ng/g) by location and focal animal. A type I, two-way ANOVA with Games–Howell post-hoc comparisons showed multiple significant differences. * *p*-value < 0.05; and *** *p*-value < 0.001.

A type one, two-way ANOVA revealed a statistically significant interaction between the effects of orangutan housing location (“Location”) and individual orangutan (“Focal”); F_(4,1425)_ = 4.033, *p*-value = 0.003, *R*^2^ = 0.006. Additionally, both “Location” and “Focal” showed a statistically significant effect on fGM values; F_Location (2,1425)_ = 57.417, *p*-value < 0.001, *R*^2^ = 0.040 and F_Animal (2,1425)_ = 672.839, *p*-value < 0.001, *R*^2^ = 0.464. A Games–Howell post-hoc comparison revealed that significant differences in concentrations of fGMs were primarily correlated with changes between the VMC and RAR2 (Figure 2). For orangutans “1149” and “B40236”, the only significant differences in fGM concentrations occurred between samples collected at the VMC and RAR2 (“1149”: *p*-value = 0.029 and “B40236”: *p*-value < 0.001), while for orangutan “B50023”, significant differences were found between RAR1 and VMC (*p*-value < 0.001) as well as RAR1 and RAR2 (*p*-value = 0.002).

**Figure 3:**
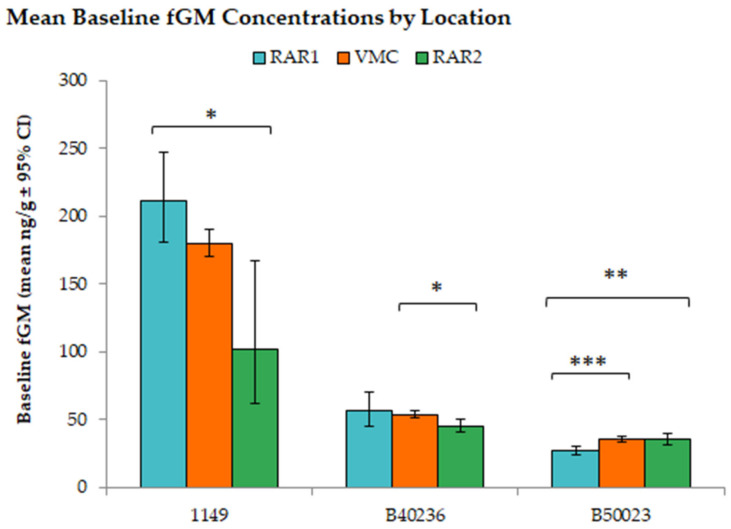
Average baseline fGM concentrations (ng/g) by location and individual. A type I, two-way ANOVA with a Games–Howell post-hoc analysis showed multiple significant differences. * *p*-value < 0.05; ** *p*-value < 0.01; and *** *p*-value < 0.001.

Baseline fGM concentrations followed the same pattern with “Location” (F_Location (2,1082)_ = 56.07, *p*-value < 0.05, *R*^2^ = 0.032) and “Focal” (F_Focal (2,1082)_ = 1167.64, *p*-value < 0.05, *R*^2^ = 0.657) significantly affecting fGM concentrations (Figure 3). Additionally, the same significant interaction between “Location” and “Focal” existed within the baseline fGM concentrations that existed in the overall fGM concentrations (F_(4,1082)_ = 5.773, *p*–value = 0.001, *R*^2^ = 0.007). Games–Howell post-hoc tests revealed nonsignificant differences between RAR1 and RAR2, but significant differences between VMC and both other locations (Figure 3). When baseline data were analyzed by individual, orangutan “1149” showed significantly different fGM concentrations between RAR1 and RAR2 (*p*-value = 0.022), orangutan “B40236” exhibited significantly different fGM concentrations between VMC and RAR2 (*p*-value = 0.012). Interestingly, orangutan “B50023” showed significantly different baseline fGM concentrations between RAR1 and RAR2 (*p*-value = 0.006) as well as RAR1 and VMC (*p*-value = 0.001).

When orangutan “1149” was excluded from analysis, a significant interaction remained between “Focal” and “Location”: F_(2,1004)_ = 4.109, *p*-value =0.017, *R*^2^ = 0.007, and “Focal” and “Location” also significantly affected overall average fGM concentrations: F_Focal(1,1004)_ = 82.08, *p*-value < 0.001, *R*^2^ = 0.017 and F_Location(2,1004)_ = 9.523, *p*-value < 0.001, *R*^2^ = 0.074. However, at the baseline level, “Location” no longer significantly affected fGM concentrations: F_(2,753)_ = 2.688, *p*-value = 0.069, *R*^2^ = 0.006. Both “Focal”, F_(1,753)_ = 176.834, *p*-value < 0.001, *R*^2^ = 0.187, and the interaction between the two fixed effects, F_(2,753)_ = 4.936, *p*-value = 0.007, *R*^2^ = 0.010, remained significant.

## 4. Discussion

The orangutans at the Oregon Zoo experienced two major intra-institutional enclosure transfer events and were housed for an extended period of time at the Veterinary Medical Center (VMC) in generic indoor holding areas, while construction of the Primate Forest habitat, located adjacent to the Red Ape Reserve (RAR), took place. Transfers to different enclosures, even within institutions, have been shown to alter fGM concentrations. For many species, inter-institutional transfers result in significant fGM elevations, or “peak” concentrations, with a return to baseline levels within a week, or sometimes several weeks (e.g., [2]). Such responses have been well documented e.g., [1,2,19,24]. Interestingly, intra-institutional transfers have not received as much attention, despite their likely more frequent occurrence. Clearly delineating what constitutes an intra-institutional transfer may not always be as straightforward as identifying an inter-institutional transfer. The latter usually necessitates that an animal is placed in a crate and transported, either on ground or by airplane, for an extended time period to a new place. Intra-institutional enclosure changes and transfers can encompass a variety of different situations, not all of them involving transportation via a crate, but nonetheless potentially stressful. For example, while not representing an actual move to a different enclosure, substantial restrictions in enclosure access, based on seasonality, are common and may result in similar physiological responses as enclosure transfers. Animals that are not adapted to the full spectrum of weather in a specific area may need to be kept in indoor housing for several months of the year to mitigate the impact of the environment. This can affect the overall physiology, including adrenal activity. For instance, giraffes at the Brookfield Zoo showed higher fGM concentrations when they were housed exclusively in an indoor habitat during the winter when compared to the summer (outdoor) habitat [24]. While our study was somewhat similar to the one conducted by Razal et al. [24] on giraffes, it differed in the extended length of time Oregon Zoo’s orangutans spent in the indoor holding areas and in the fact that it involved a physical transfer (crated transport across the Zoo) to an entirely unfamiliar enclosure space at the VMC. 

Since the VMC is designed for mostly short-term holding of a wide variety of species, it is challenging to customize available spaces for any particular species that may be housed longer-term. While intense effort went into preparing several connected indoor enclosures specifically for orangutans by adding lots of species-specific enrichment items, bedding options, and climbing structures, the available modifications were nevertheless limited due to the lack of an outdoor area and substantially reduced enclosure height. Previous studies, including one on orangutans at the Oregon Zoo conducted in 2000 [25], showed increased active behavior and decreased salivary cortisol concentrations when an enclosure was specifically designed to meet the species’ needs. Unfortunately, our study did not include behavioral monitoring due to a lack of access for observers while the animals were housed at the VMC. This access limitation was primarily due to COVID-19 restrictions, but also due to very limited space available around the VMC enclosures and a lack of camera access. However, our fGM monitoring results are similar to those found in Tingey [25], with two of the three orangutans showing decreased overall fGM concentrations once they were moved back to the RAR facility designed for orangutans. 

Surprisingly, none of the intra-institutional transfers between the VMC and the RAR resulted in a significant peak in fGM concentration for any orangutan. As mentioned previously, inter-institutional transfers typically result in significantly increased glucocorticoid concentrations, and are usually considered to be intense stressors, and thus used for providing a biological validation of the fGM analysis methodology [26]. Interestingly, and in apparent contradiction to our findings on orangutans, a previous study on black rhinoceros (*Diceros bicornis*) showed distinct peaks in fGM concentrations in three out of four monitored individuals when transferred to a new enclosure within the same institution [27]. These peaks were present despite training protocols designed to help the black rhinoceroses acclimate to their transfer crates. However, Göttert et al. [27] did conclude that the training protocols and intra-institutional transfer caused the stress response to be on the “lower end” (pg. 26, [27]) of reported black rhinoceros stress responses. Therefore, these results may actually support our hypothesized finding of a possible mitigation of transport stress due to substantial pre-transport training in the orangutans which may have led to a lack of a significant fGM response. 

There are, of course, a variety of possible explanations for the less-stressful nature of intra-institutional transfers such as the aforementioned pre-transport training, much shorter transport distance and duration when compared to inter-institutional transfers, consistent familiarity with the animal care staff, long-term established positive keeper-animal relationships (KAR), and potential previous familiarity with the transfer enclosure, especially if repeat transfers occur (like in our case with the transfer back to the RAR). In our study, all three orangutans were voluntary participants in multiple weeks of training sessions revolving around minimizing the stress of transferring them from one habitat to another. Care staff worked with the orangutans and provided them with opportunities to become comfortable with the transfer crates prior to the move. This training may have sufficiently minimized any potentially stressful effects of the transfer. Operant training has been shown to reduce stress during capture and transport for a multitude of species (e.g., *Saguinus labiatus* [28], *Tragelaphus eurycerus* [29,30], and *Leptoptilos crumeniferus* [31]) and positive reinforcement training conducted prior to potentially stressful situations has been correlated with positive outcomes such as less dramatic spikes in salivary cortisol in orangutans following medical procedures [32] and increased affiliative behaviors in ring-tailed lemurs (*Lemur catta*) [33] and vervet monkeys (*Chlorocebus aethiops*) [34] during periods of social isolation. However, a more involved study comparing fGM concentrations and behavior expression across various training regimes would be a great future project to better identify the most effective and impactful training methods to prepare orangutans for transfers and significant habitat changes. 

It could also be possible that the long-term positive relationships between the care staff and the animals helped to mitigate potential physiological responses. A general study by Hosey and Melfi [35] suggested that the keeper-animal bond (i.e., positive KAR) may bring positivity and less stressful interactions to both the keepers and the animals in their care. In intra-institutional transfers, the relocated individual’s original care team remain involved and this level of familiarity may provide comfort and stability for the animals in their new facilities. Furthermore, a study specific to orangutans showed decreased aggression and hiding behaviors when orangutans were interacting with familiar care staff, indicating increased contact and familiarity between care staff and orangutans could have an overall positive impact on orangutans [36,37]. The relationship between care staff familiarity and positive impacts has also been demonstrated in other species. For instance, more established care staff to animal relationships were associated with increased willingness to embrace new surfaces [38] and successful task completion [39] in Asian elephants (*Elephas maximus*). While a more in-depth study is needed to quantify the effect of increased contact with care staff on the physiological changes associated with dramatic habitat differences, we believe that the intense care and positive relationships provided by our care staff may have helped to buffer the orangutans from the potential stressful effects of the two moves and housing changes. 

Finally, the lack of novelty associated with RAR2 may have dampened the expected spike in fGM concentrations for the second transfer. Both RAR1 and RAR2 were the same physical habitat and there were no major alterations to RAR during the orangutans’ time at the VMC (construction was on the chimpanzee habitat adjacent to RAR). Habitat novelty has been successfully used as an experimental stressor to increase glucocorticoids in many biomedical studies (e.g., *Mus musculus* and *Rattus norvegicus* [40,41]). In inter-institutional transport studies, it is difficult to discern the direct cause of glucocorticoid spikes (e.g., was it novelty, handling, crate-time, etc., that caused the increased fGM levels) and these stressors are usually discussed holistically (see Dickens et al. [42]). However, intra-institutional studies may allow for more controlled comparisons to potentially identify causal relationships between increased glucocorticoid responses and specific aspects of the transfer process. 

Apart from the unexpected lack of a significant peak in fGM concentrations directly following the intra-institutional transfers, our study showed elevated overall fGM concentrations when the orangutans were housed at the VMC, although the elevation was nonsignificant for one of the orangutans “B50023”. Interestingly, this overall pattern did not persist when only baseline fGM concentrations were observed. For orangutans “1149” and “B40236”, average baseline fGM concentrations were highest when they were housed in RAR1, although they varied insignificantly from samples collected at the VMC. Unlike the other two orangutans, orangutan “B50023” had the lowest average baseline fGM concentration at RAR1 and these concentrations did significantly differ from samples collected at the VMC. However, “B50023” did not show the significant decrease in baseline fGM concentrations between VMC and RAR2 that was observed in the other two orangutans. This could, in part, be due to a change in reproductive status. “B50023” was on birth control when the study started and was “taken off” birth control in December 2019, to comply with a breeding recommendation for her and orangutan “B40236”. While previous research did not find significant differences in orangutan glucocorticoid expression based on reproductive cycle-stage of reproductively active individuals [6], the differences associated with moving from non-reproductive to reproductively active may have influenced the fGM concentrations as seen in several other species (e.g., *Panthera leo* [43], *Acinonyx jubatus* [44], and *E. maximus* [45]). Prior to the birth control removal, orangutan “B50023” did not experience any cyclic progesterone metabolite patterns and fecal progesterone metabolite concentrations were at baseline levels. After orangutan “B50023”’s birth control regime was stopped, she had low and sporadic estrus cycles until March 2020 whereafter she began to cycle consistently.

It is interesting to note the uniqueness of each orangutan’s fGM response to the enclosure changes. Orangutan “1149” exhibited the highest overall average fGM concentrations and seemed to show more pronounced variability to location changes. This may have been in part due to her advanced age, differing fecal consistency (indicating possible metabolic differences related to age), and potential species differences. At the time this study was conducted, according to the studbook, orangutan “1149” was the oldest orangutan in North America at just under 61 years of age. Previous research has reported contradictory evidence of the effect of age on fGM concentrations. Takeshita et al. [5] showed no significant correlations between age and fGM concentrations in their study of orangutans but Weingrill et al. [6] documented a significant positive effect of age on female orangutan fGM concentrations. Age has also been correlated with elevated glucocorticoid excretion in other great apes (e.g., chimpanzee urinary cortisol [46]). Further study is needed to establish whether this may be consistently the case for orangutans as well.

It also must be noted that orangutan “1149” was the only Sumatran orangutan (*P. p. abelii*) included in the study and housed at the Oregon Zoo—the other individuals were Bornean orangutans (*P. p. pygmaeus*). Sumatran orangutans and Bornean orangutans differ in their lifestyles, with Sumatran orangutans maintaining the highest level of sociability and the lowest reaction to social stressors [6,47]. Interestingly, fGM results for orangutan “1149” did not reflect these previous findings, as she displayed higher variability in fGM concentrations, and both overall and baseline fGM concentrations were found to be significantly higher than in the two Bornean orangutans. This differing pattern may indicate that ‘age’ has a stronger influence than ‘species’ on the observed differences in fGM concentrations. Additionally, orangutan “1149” was the only orangutan included in our study that was wild-born. She arrived at the Oregon Zoo in 1961 at approximately 1 year of age after having been collected for the animal trade and confiscated. A study by Muehlenbein et al [48], showed lower fGM concentrations in orangutans habituated to human contact. While orangutan “1149” was maintained in captivity for almost her entire life, it is possible that her wild-born background contributed to her increased fGM concentrations. A collaborative meta-study of different species of orangutans housed in multiple institutions would help to further explore the relationship between age, species, and fGM concentrations.

While age and species may have impacted the overall fGM concentrations, changes in fecal consistencies may also have affected fGM concentrations. As part of the Oregon Zoo’s ongoing endocrine monitoring program, the fecal samples were analyzed for overall water content in an effort to minimize variability in concentrations due to differences in water content. Both orangutans, “B40236” and “B50023”, maintained consistent fecal water content of ~70%, while orangutan “1149” had significantly drier feces with approximately 48% water content. On 1 July 2020, orangutan “1149” was prescribed Metamucil to assist in improving her fecal consistency, and her overall water content increased to 64%. Variable water content can directly impact measured fGM concentrations as water dilutes the amount of fecal content being weighed for comparison [49]. One way to mitigate the variability is to dry the fecal samples prior to weighing. However, if fecal water content remains consistent within an individual, or is accounted for by periodic water content tests, the patterns revealed by fecal hormone analyses can be compared. Orangutan “1149” had elevated overall fGM concentrations throughout the study and this trend persisted even when water content was accounted for, indicating a different cause of elevation, such as age, health related differences, or species differences. 

Our study had several obvious limitations, in part due to the opportunistic nature of the study. Our findings are based on a very limited sample size of only three individuals, we had unequal samples sizes across “treatments”, and we were unable to collect additional data, such as behavior observations which could have added to our understanding of the variation in hormonal responses. Nevertheless, we believe that, given the endangered status of both orangutan species and the unique transfer and housing situation encountered by our orangutans, the presented data can provide an important contribution to our understanding of orangutans’ adrenal responses in human care, adding to the body of existing knowledge on this topic and also provide a starting point for future studies.

## 5. Conclusions

This study used long-term fGM monitoring to highlight the impact of extended housing changes and repeat enclosure transfers on the adrenal activity of three orangutans. We found differences in overall fGM concentrations between VMC and RAR in all three orangutans, with increased hormone values when orangutans were housed at the VMC, although there was substantial individual variation in responses. This pattern was not replicated when baseline fGM concentrations were evaluated. For two orangutans, the highest average baseline fGM concentrations occurred at RAR1 while the third individual experienced the highest average baseline fGM concentrations at the VMC. Overall fGM concentrations quickly declined when individuals returned to their old habitat for two of the orangutans and a minorly declined in one individual; however, changes in reproductive status concurrent to moving this individual back to RAR may have affected her adrenal response. Overall, these results appear to highlight the need to minimize the duration of time orangutans are housed in a temporary holding facility. While the occasional off-habitat housing is unavoidable (e.g., due to construction), and the overall benefit may be significant (e.g., a more species-appropriate habitat through construction, as well as not sending animals to other facilities and exposing them to potentially significant inter-institutional transport stress), planning should prioritize minimizing the amount of time spent away from the species-specific habitat. 

Secondly, this study proposed the importance of pre-transfer training and an existing close and positive keeper animal relationship to help mitigate potentially stressful events, including a long-term stay and acclimation to indoor holding facilities. Allowing for sufficient staff time to engage in trust building, ample training, and all aspects necessary for a positive and respectful keeper animal relationship must be an integral part for any great ape program.

Lastly, this study provided more evidence in support of significant individual variation in fGM responses. These individual differences may, in part, be due to age, gender, life history, individual history, and also temperament. An increased understanding of the different factors contributing to individual variation is imperative for accurate interpretation of endocrinological data. More studies are needed to investigate these differences systematically.

While our findings point to some interesting avenues for further study, we caution any extrapolation of the results due to the significant individual variation in fGM responses. It is important to monitor each animal individually and take into consideration possible age, gender, species, and temperament related differences when conducting studies of adrenal activity associated with enclosure changes and animal transfers. 

## Figures and Tables

**Table 1:** Orangutan identification and demographic information.

Orangutan ID	Date of Birth	Gender	Species
B50023	23 April 2001	Female	*P. p. pygmaeus*
B40236	23 January 2006	Male	*P. p. pygmaeus*
1149	Unknown, 1960	Female	*P. p. abelii*

**Table 2:** Orangutan descriptive fecal glucocorticoid metabolite (fGM) data by individual and location. “Overall *n*”: all fecal samples collected over the observational period. “Baseline *n*”: number of fecal samples after the removal of all fecal samples >2 standard deviations (SD) above the mean.

Focal	Location	Overall *n*	Mean ng/g (± SD)	Baseline *n*	Mean ng/g (± SD)
B50023	RAR1	29	34.54 (±20.05)	25	28.53 (±7.92)
	VMC	418	64.30 (±55.05)	310	39.46 (±17.39)
	RAR2	59	57.00 (±46.73)	45	38.58 (±14.69)
B40236	RAR1	25	80.38 (±67.68)	23	63.12 (±29.37)
	VMC	421	86.00 (±57.81)	307	57.59 (±19.58)
	RAR2	58	59.24 (±31.84)	49	48.43 (±16.87)
1149	RAR1	10	215.78 (±45.30)	10	215.78 (±45.30)
	VMC	402	277.44 (±190.49)	311	199.35 (±81.28)
	RAR2	12	146.14 (±99.57)	11	125.46 (±72.57)

## Data Availability

The data presented in this study are available upon request from the corresponding author. The data are not publicly available due to the confidential nature of health data at the Oregon Zoo.
